# Interferometer-based structured-illumination microscopy utilizing complementary phase relationship through constructive and destructive image detection by two cameras

**DOI:** 10.1111/j.1365-2818.2012.03604.x

**Published:** 2012-06

**Authors:** L Shao, L Winoto, DA Agard, MGL Gustafsson, JW Sedat

**Affiliations:** * Keck Advanced Microscopy Laboratory, Department of Biochemistry and Biophysics, University of CaliforniaSan Francisco, California, U.S.A.; † Howard Hughes Medical Institute, Chevy ChaseMaryland, U.S.A.; ‡ Janelia Farm Research Campus, Howard Hughes Medical InstituteAshburn, Virginia, U.S.A.

**Keywords:** Image reconstruction, interferometer, I^5^S, optical transfer function, structured-illumination, widefield fluorescence microscopy

## Abstract

In an interferometer-based fluorescence microscope, a beam splitter is often used to combine two emission wavefronts interferometrically. There are two perpendicular paths along which the interference fringes can propagate and normally only one is used for imaging. However, the other path also contains useful information. Here we introduced a second camera to our interferometer-based three-dimensional structured-illumination microscope (I^5^S) to capture the fringes along the normally unused path, which are out of phase by π relative to the fringes along the other path. Based on this complementary phase relationship and the well-defined phase interrelationships among the I^5^S data components, we can deduce and then computationally eliminate the path length errors within the interferometer loop using the simultaneously recorded fringes along the two imaging paths. This self-correction capability can greatly relax the requirement for eliminating the path length differences before and maintaining that status during each imaging session, which are practically challenging tasks. Experimental data is shown to support the theory.

## Introduction

We reported earlier a resolution-enhancing scheme for three-dimensional (3D) widefield fluorescence microscope, I^5^S (Shao *et al.*, 2008), that greatly improves the resolution of optical microscopy to nearly 100 nm in all three dimensions. It combines structured-illumination microscopy (SIM; Heintzmann & Cremer, 1999; Gustafsson *et al.*, 2000; Gustafsson, 2000; Gustafsson *et al.*, 2008) with an interferometer using two opposing objective lenses (Hell & Stelzer, 1992; Gustafsson *et al.*, 1999). In SIM, the sample is illuminated by a periodic pattern, which mixes with the otherwise undetectable high-resolution information and, in spatial frequency space, moves it into the passband of the microscope in the form of moiré fringes. The down translated high-resolution information is then computationally recovered to produce an image with extended resolution (Heintzmann & Cremer, 1999; Gustafsson *et al.*, 2000; Gustafsson, 2000; Gustafsson *et al.*, 2008). SIM can double the conventional resolution in all three dimensions, resulting in a resolution of about 100 nm laterally and 300 nm axially (Gustafsson *et al.*, 2008). I^5^S is an extension of SIM in which a second objective lens is used and placed on the opposite side of the sample, and the two apertures are combined interferometrically (Hell & Stelzer, 1992; Gustafsson *et al.*, 1999; Shao *et al.*, 2008). As a result, the axial resolution is greatly improved both because the set of light-gathering angles of the microscope is enlarged and because the highest axial spatial frequencies in the illumination pattern are increased due to the interference of the illumination beams coming from the two opposing objectives. An isotropic 100-nm-scale resolution is achieved by this technique in all dimensions (Shao *et al.*, 2008).

**Fig. 1:**
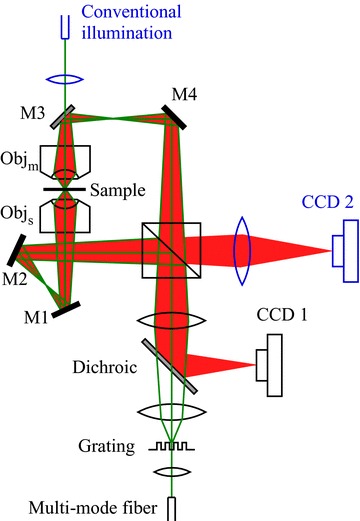
The schematic drawing of an I^5^S microscope. The illumination light passes first through a transmission grating, which diffracts it into three beams (green lines), and then through a beam splitter, which splits each beam and directs three beams to each of the two opposing objective lenses. The same beam splitter combines the two beams of emission light (red) from the sample onto the camera. Normally, only one camera (CCD 1) is used to record the emission light from one of the two beam splitter exit ports. Here we introduced a second imaging path to record on CCD 2 the emission light from the other exit port. The movable objective lens can be positioned in X, Y and Z with respect to the stationary objective lens. Mirrors M3 and M4 can be translated together to adjust the path length difference. The grating can be rotated and laterally translated to control the orientation and lateral phase of the illumination pattern. Mirrors M3 is made partially transmissive to let conventional illumination come in from there so that I^5^S detection OTF can be measured.

**Fig. 2:**
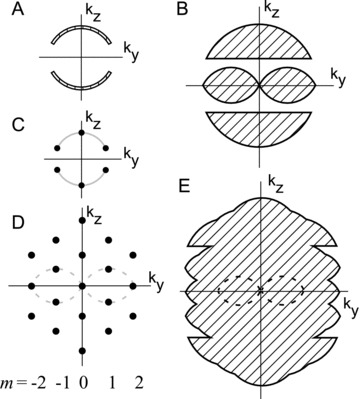
Principles of I^5^S explained in frequency space. (A) The generalized pupil function (GPF) of an I^5^S/I^5^M microscope, i.e. the portions of the wavefront detectable through the two objective lenses. In a conventional widefield microscope, only one of the two shells is detectable. The support (i.e. region of nonzero value) of the intensity detection OTF, which is the autocorrelation of the GPF, is shown in (B). In comparison, the detection OTF of a conventional microscope is just the middle region. (C) The six dots represent the six collimated illumination beams being incident upon the sample. Their interference creates a 19-component illumination intensity pattern (D) organized into five vertical lines corresponding to *m*=−2 to 2. (E) The effective I^5^S OTF support is shown in k_y_–k_z_ cross-section. As a reference, the conventional OTF support is drawn in dashed contour in (E).

**Fig. 3:**
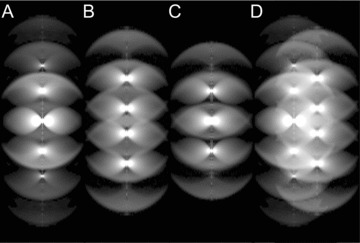
Experimentally measured and rotationally averaged I^5^S OTFs *O_m_* of Eq. (2) for *m*= 0, ±1 and ±2 (A—C). (D) Demonstrates that *m*= 0 and *m*=±1 components partly overlap when they are placed where they belong in frequency space.

In I^5^S, a collimated laser illumination beam is diffracted by a transmission phase grating and the central three diffraction orders are split by a beam splitter, resulting in six mutually coherent illumination beams being incident on the sample, one triplet of beams passing through each objective lens (Fig. 1). The illumination pattern that results from the interference of these six beams consists of 19 frequency components (Fig. 2D) and can be expressed as (Shao *et al.*, 2008)


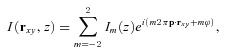
(1)

where **r**_*xy*_ represents the lateral coordinates (x, y), **p** is the lateral component of the wave vector of each beam not coincident with the optical axis *k*_*z*_(or the side beams), *I_m_*(*z*) is the *m*th-order axial illumination pattern in real space corresponding to the *m*th axial row of illumination frequencies shown as dots in Figure 2D and 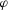 is a phase angle (Gustafsson *et al.*, 2008; Shao *et al.*, 2008). Given the form of the illumination pattern in Eq. (1) and the condition that the axial position of the illumination pattern is fixed in relation to the focal plane of the microscope as the sample is refocused during 3D acquisition, the Fourier transform 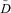 of an observed 3D image *D* can be expressed as follows (Gustafsson *et al.*, 2008; Shao *et al.*, 2008):


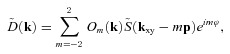
(2)

where 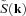 is the sample information, *O_m_* (Figs 3A–C) is the *m*th-order effective optical transfer function (OTF) obtained by convolving the detection OTF with the axial Fourier transform of *I_m_*. The axially extended support, or the nonzero valued region, of *O_m_* in I^5^S is attributed to both the high axial illumination frequencies (Fig. 2D) and a detection OTF with two axially extended support regions (or the sidebands; Fig. 2B) in addition to the usual OTF support region (or the central band) of the single-objective microscopy (Gustafsson *et al.*, 1999). Equation (2) shows that I^5^S improves the resolution laterally via frequency shift of the sample information and axially via the extended support in the effective OTFs.

### Image reconstruction and path length difference

The raw data of SIM, including I^5^S, is the summation of five components (Eq. (2)). These components can be separated given a series of images taken with five different lateral pattern's phases (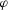 in Eqs (1) and (2); Gustafsson *et al.*, 2008; Shao *et al.*, 2008). Because the lateral pattern in our implementation is one-dimensional (1D), the same pattern needs to be applied multiple times with different angles (i.e. **p** in Eqs (1) and (2) with the same magnitude but different orientations) so as to isotropically extend the lateral resolution. A final image with 100 nm resolution in all three dimensions (Fig. 2E) is reconstructed by positioning all the separated information components back to their original locations in frequency space and then correcting for amplitude differences via a generalized Wiener filter (Gustafsson *et al.*, 2008; Shao *et al.*, 2008).

For I^5^S reconstruction to work, it is required that the raw image is acquired under the same conditions as the measured effective OTFs. In I^5^S or any interferometric set-up, one condition prone to variation in different imaging sessions is the path length difference of the interferometer loop. Before every experiment, significant time must be spent on digitally adjusting this path length difference to reach zero, which gives the maximum axial fringe contrast. Even if this preimaging adjustment is performed very accurately, instrumental drift will have caused different path length differences, i.e. different imaging conditions, in the actual data set and in the data set taken for the OTF determination, resulting in axially ringing artefacts in the reconstruction because of the unmatched OTF used. To make I^5^S easier to use, we need a scheme that can relax this very stringent requirement on path length difference consistency without inducing artefacts.

Another disadvantage of the current I^5^S set-up is that only the imaging interference fringes at one of the beam splitter's two exit ports are recorded, meaning half of the emission light is discarded. Capturing both halves of the emission light simultaneously would significantly improve the signal-to-noise ratio. Furthermore, because of the complementary phase relationship between the fringes at the two beam splitter exits (to be discussed next), the actual path length difference within the interferometer loop can be estimated from a pair of 3D images simultaneously acquired through the two exits. With this quantity known, one can then either choose a matching set of OTFs from a library of OTFs or, in a more attractive solution, synthesize a set of OTFs that can account for the estimated path length difference, and use those to reconstruct the data set. As a result, the requirement for achieving a specific path length difference for each experiment can be relaxed.

## Path length difference estimation

**Fig. 4:**
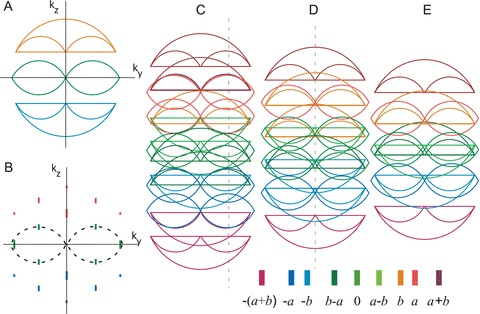
The maps of phase values in I^5^S detection OTF (A), illumination structures (B) and the effective OTFs for *m*= 0 (C), ±1 (D) and ±2 (E). The reason why most illumination components are stretched axially compared to Figure 2D was explained in detail in earlier publications (Gustafsson *et al.*, 2008; Shao *et al.*, 2008). The regions coloured differently correspond to different phases equal to various linear combinations of *a* and *b*, where *a* and *b* are the phase differences caused by the path length difference in the interferometer loop for the excitation and emission wavelengths, respectively. In reality, the dashed lines in (C) and (D) coincide, i.e. they represent the same lateral frequency.

First, we discuss how the path length difference can be estimated from an I^5^S data set. Given a known path length difference δ, the detection OTF of I^5^S can be divided into three regions based on different phases: *b*, 0 and −*b*, where *b*= 2*πδ*/λ_em_ and λ_em_ is the emission wavelength (Fig. 4A). These values are because of the fact that the detection OTF is an autocorrelation of the generalized pupil function (GPF; Fig. 2A) and the relative phase difference between the two shells of GPF (i.e. the two wavefronts collected separately by the two objective lenses) is *b*. Likewise for the illumination components (Fig. 4B), three groups can be distinguished based on the components’ phases: *a*, 0 and −*a*, where *a*= 2*πδ*/λ_ex_ and λ_ex_ is the excitation wavelength. Because the effective I^5^S OTF *O_m_* is the convolution of the detection OTF with the *m*th illumination order 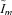 (Eq. (2) and Fig. 3) and phases of the operands add in convolution, the phases in different regions of I^5^S effective OTFs are various additive combinations of 0, ±*a* and ±*b*. The colour-coded maps of phase in these OTFs are shown in Figures 4C–E.

**Fig. 5:**
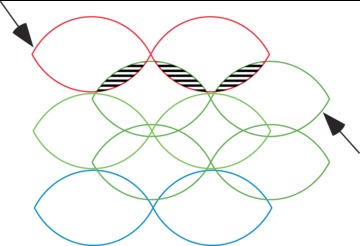
When the *m*= 1 component (Fig. 4D) is placed where it belongs in frequency space, it partly overlaps with the *m*= 0 component as indicated by the shaded areas. Shown here are only the regions because of the convolution of central band of the detection OTF (Fig. 4A, green) and the central three and two frequencies of the *m*= 0 and 1 illumination structure, respectively (Figs 2D and 4B).

Because the *m*th-order component of the I^5^S raw data is a subregion of the sample's Fourier transform—corresponding to the support of *O_m_* shifted by *m***p** (Eq. (2))—multiplied by *O_m_*, this component's phase map is the addition of the sample's and the OTF's phases. Furthermore, components of different orders partially overlap with each other in frequency space (Fig. 3D). For example, the central line of the *m*= 1 component (the dashed line in Fig. 4D) should be placed **p** distance (Eqs (1) and (2)) from the central line of the *m*= 0 component; i.e. to the dashed line in Figure 4C. If we ignore the portions of the effective OTFs derived from the sidebands of the detection OTF for the moment and move the *m*= 1 component to its original position in frequency space, as illustrated in Figure 5, the green coloured part of the *m*= 1 component partially overlaps with the red coloured part of the *m*= 0 component (shaded areas in Fig. 5). Within this overlap, the raw image's *m*= 0 and 1 components at a certain frequency **k** are related by


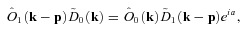
(3)

where 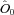 and 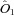 are OTFs that are acquired with zero path length difference and 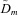 is the *m*th-order component 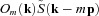 separated from Eq. (2) (the zero path length difference requirement on 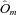 can be satisfied even for OTFs acquired with nonzero path length difference, to be discussed in *OTF separation and synthesis*). Linear regression can then be applied to solve Eq. (3) for *a* using all the frequencies within the overlap. This seems to be a straightforward method to estimate the path length difference, except that it requires a clean overlap between only the two central band-derived regions of the *m*= 0 and 1 components pointed to by the arrows in Figure 5. Unfortunately, superimposed on those two central band-derived regions are other signals derived from the sidebands of the detection OTF (Figs 3 and 4C–E), which make Eq. (3) invalid. Hence, we first need a solution for obtaining the uncontaminated overlaps as depicted in Figure 5 before the path length difference can be estimated using Eq. (3).

### Estimation based on complementary phase relation

It can be proved (see Appendix) that in I^5^S, when the two emission wavefronts collected from the two objective lenses are coherently combined at the beam splitter, the phase of the interference fringes at the primary exit port (i.e. the illumination entry port, Fig. 1) of the beam splitter is exactly π different than that at the other exit port (the secondary exit). This results in the following relation between the detection OTFs acquired at the primary and the secondary exits: when the phases are ±*b* in the sidebands of the former, the phases are ±(*b*+π) in the sidebands of the latter. Then if I^5^S effective OTFs are acquired through the two exits, the same π phase difference exists between the two sets of OTFs in those regions because of the convolution of the illumination components with the sidebands of the detection OTF. Furthermore, as an image in frequency space is a multiplication of the object's Fourier transform by the OTF, this π phase difference in those sideband-derived regions is also there between two I^5^S data sets acquired simultaneously through the two exits for any sample. Consequently, the addition (or subtraction) of these two data sets results in only the central (or side) band-derived regions being nonzero in frequency space, provided that correct intensity normalization between the two images has been applied. If the addition image is used for extracting the overlap shown in Figure 5, the overlap would not be contaminated by the superimposed sideband-derived signals and therefore, the path length difference can be cleanly estimated as described in Eq. (3).

**Fig. 6:**
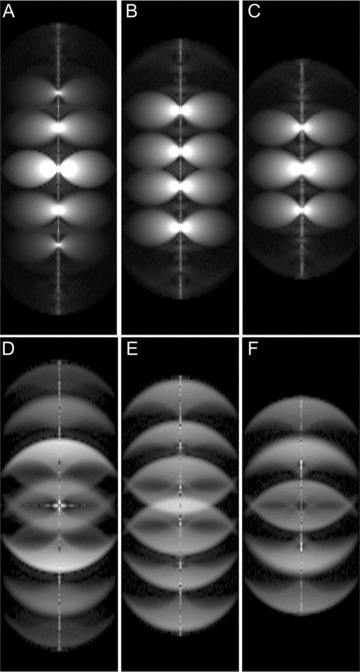
The effective I^5^S OTFs were acquired through the primary and secondary beam splitter exit and rotationally averaged, and their additions and subtractions, for *m*= 0, ±1 and ±2, are shown in (A)–(C) and (D)–(E), respectively. The regions derived from the sidebands of the detection OTF are mostly missing in the additions, although those from the central band are missing in the subtractions, as theoretically predicted.

To implement the above idea, a second imaging path was added to I^5^S to capture the interference fringes through the secondary exit of the beam splitter (CCD 2 in Fig. 1). A pair of I^5^S point spread functions (PSF) was acquired simultaneously by the two CCDs and the rotationally averaged effective OTFs were generated (Gustafsson *et al.*, 2008; Shao *et al.*, 2008). The summations and subtractions of these two sets of OTFs are shown in Figure 6, which, as expected, contain mostly the subregions derived only from the central band and sidebands of the detection OTF, respectively.

To estimate the path length difference in each data set, a pair of raw data sets was acquired simultaneously by the two cameras. The image pair was then computationally aligned with each other to compensate the residual misalignment between the imaging paths. The misalignment, mainly because of lateral shift, rotation and magnification, only needs to be calibrated once every few months using scattered beads samples. The result of the calibration, a two-dimensional affine transform matrix, was saved for aligning the image pairs in subsequent experiments. The intensity ratio between the image pair after background subtraction was also calibrated and saved for future use. After alignment and intensity correction, each raw image was separated into the five information components (Eq. (2)), each corresponding to a different *m* of Eq. (2) (Shao *et al.*, 2008), and the *m*= 0 and 1 components of the images acquired by one camera were added to their counterparts acquired by the other, resulting in the new *m*= 0 and 1 components with little sideband-derived contamination. The overlap between the red subregion of the *m*= 0 component and the green subregion of the *m*= 1 component (Fig. 5) was then extracted and used for the estimation of the path length difference based on Eq. (3).

## OTF separation and synthesis

Given the estimated path length difference, we still need a scheme for image reconstruction to take advantage of it. Here we introduce such a scheme based on the fact that I^5^S effective OTFs are the convolution of the detection OTF *O* with the 1D axial illumination structures 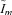 (Eq. (2)). Suppose a measured I^5^S OTF can be decomposed into *O* and 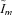. Once the path length difference is estimated from a raw image, then it is trivial to modify the phases of *O* and 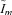 by multiplying 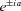 to the 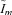 components of nonzero phases (Fig. 4B) and 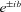 to the sidebands of *O*(Fig. 4A). The phase-modulated *O* and 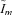 can then be convolved together to synthesize a set of OTFs that can account for the path length difference specific to the raw image.

To separate I^5^S OTFs into two convolving parts, one of the parts has to be known. There is a way to measure the detection OTF and I^5^S effective OTFs almost simultaneously as long as the mirror M3 in Figure 1 also transmits partially (20% is what we used) so that a conventional illumination path can be introduced through that mirror. With this new set-up (Fig. 1), the 3D I^5^S effective PSF and detection PSF can be acquired in an interleaved manner by alternating between the conventional and the structured-illumination path at each defocus step. We can then obtain rotationally averaged I^5^S effective OTFs and detection OTF from these PSFs.

The OTF separation problem is formulated as solving for 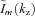 in 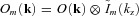 given *O_m_*(**k**) and *O*(**k**). Because 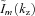 is a 1D function, this problem is equivalent to solving it in a series of 1D deconvolution problems 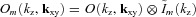 for each lateral frequency **k**_xy_. Linear convolution can be transformed into a circular convolution, which can then be formulated as a vector (in this case the unknown 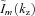) being multiplied by a cyclic matrix, if the vectors have a sufficiently short support or are padded with enough zeros (Bertero & Boccacci, 1998, Chapter 2.7). A stack of linear equations, one for each **k**_xy_, can thus be formulated into an overdetermined system for solving 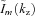. The axial illumination structures thus solved and the measured detection OTF are still modulated by an unknown phase because of the unknown path length difference during data acquisition. To estimate this path length difference, the same summation–subtraction and overlap extraction steps described above can be applied to the I^5^S effective OTFs measured by the two cameras. The estimated path length difference can then be removed from *O* and 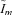 and, therefore, we can synthesize the I^5^S effective OTFs with zero path length difference (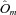 in Eq. (3)) by convolving the zero phase-modulated *O* and 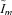, which are needed to correctly estimate path length difference in each I^5^S data set (Eq. (3)). For each of the three lateral pattern orientations, data-specific I^5^S effective OTFs are synthesized based on the estimated path length difference as described above and then used in reconstructing the normal I^5^S data set acquired through the primary beam splitter exit.

## Results

**Fig. 7:**
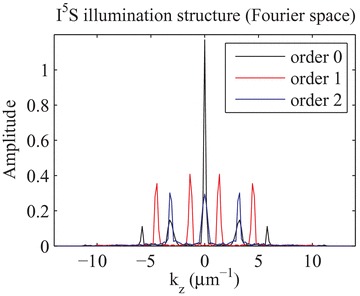
I^5^S illumination structure axial profiles in frequency space, obtained by deconvolving measured I^5^S detection OTF from the I^5^S effective OTFs. Black, red and blue curves correspond to order *m*= 0, ±1 and ±2 components, respectively.

Figure 7 shows the results of separating I^5^S illumination structures from the measured I^5^S effective OTFs as discussed above. The three curves in Figure 7 are the amplitudes of I^5^S illumination structure versus *k_z_* in frequency space for *m =* 0, ±1 and ±2 (Eqs (1) and (2)).

**Fig. 8:**
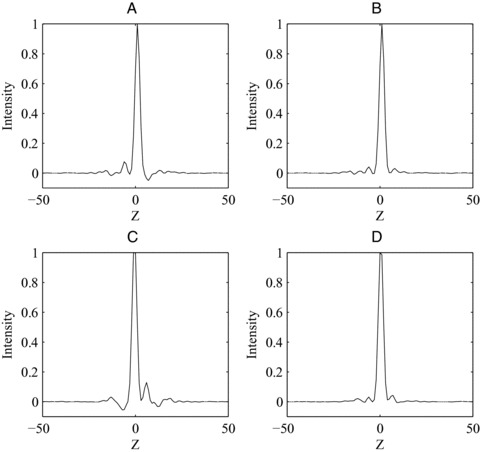
Results showing the effects of path length difference estimation and correction. (A) and (C) The axial profiles of I^5^S reconstruction of a single bead, imaged with intentional path length differences estimated to be 0.15 and −0.9 radians, respectively. (B) and (D) Results using synthesized OTFs based on the estimated path length differences corresponding to (A) and (C), respectively. There are significantly less ringing artefacts in (B) and (D) than in (A) and (C).

Two pairs of dual-camera I^5^S images of a single-bead sample were acquired. Nonzero path length differences were intentionally introduced when acquiring these data sets. The reconstructions using a standard set of effective OTFs taken with little path length difference present show the expected ringing artefacts in the axial intensity profile through the bead centre (Figs 8A and C). The different polarities of the asymmetry in those plots indicate either positive or negative path length difference in the loop. For each data set, we performed phase difference estimation (the results are 0.15 and –0.9 radians for data set shown in Figs 8A and C, respectively) and data set-specific OTF synthesis as described above, and then reconstructed the images acquired through the primary beam splitter exit using the synthesized OTFs. The results are also displayed as axial intensity profiles in Figures 8B and D, which exhibit much less ringing than Figures 8A and C.

## Discussion

We introduced a method to estimate the path length difference present in each I^5^S data set with the assistance of a second camera recording fluorescence emission's interference fringes through the secondary exit of the beam splitter. The estimation method exploits the π phase difference relationship between the imaging interference fringes at the primary and secondary beam splitter exits and the well-defined Fourier space phase maps for all the information components constituting an I^5^S data set. Another way to think about the π phase difference is to recall that when the primary beam splitter exit has constructive interferences information, the new secondary beam splitter exit will have destructive interferences information. With the estimated phase difference, a data set specific set of OTFs can then be synthesized, because I^5^S effective OTFs are separable into a detection OTF convolving with the axial illumination structures, and used for reconstructing the data set. We have used a single bead imaging experiment to prove the principle. Reconstruction using a synthesized, as opposed to a standard, set of OTFs successfully removes most of the axial ringing artefacts that are because of an unknown path length difference. One major difficulty of I^5^S or any other interferometric microscope is the strict requirement on path length difference adjustment. Relaxing this requirement will significantly enhance the real-world applicability of I^5^S to biology. The basic principle demonstrated in this paper lays the groundwork for a scheme that can achieve such a goal. It does not, however, affect other factors limiting the application of I^5^S to biology, including the requirement of refractive index matching among the mounting medium, the immersion medium and the sample itself, and the requirement that the two objective lenses are closely matched in their optical properties. In our demonstration, the fluorescent bead was mounted in the same medium as the high refractive index (1.51) immersion medium.

As mentioned earlier, the image pair recorded on two cameras can also be used simply for improving the signal to noise. This can be accomplished by including in the generalized Wiener filter (Gustafsson *et al.*, 2008) the additional terms corresponding to the images and OTFs acquired by the second camera once the image pair is aligned.

## Figures and Tables

**Fig. 9:**
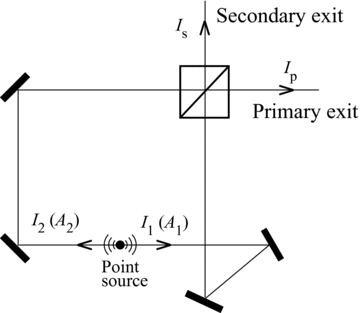
Proof of π phase difference between the interference fringes at the primary and the secondary beam splitter exits. See Appendix for details.

## Appendix

Here we prove the π phase difference between the interference fringes at the primary and secondary exit port of the beam splitter. Two parts of a wavefront with intensities *I*_1_ and *I*_2_ combine at the beam splitter after traversing the two arms of the interferometer. Each of them is split into halves by the beam splitter, with one-half transmitting through and the other half reflected by the beam splitter (Fig. 9). The reflected half of wavefront 1 either has phase φ1 +π, where φ1 is the phase before the split, if the reflection occurs at the glass–glue interface, or φ1 + 2θ if the reflection occurs at the glue–glass interface according to the Stokes relations (Hecht, 1998, Chapter 4.10), where θ is the phase delay because of the glue. The reflected half of wavefront 1 interferes at the primary exit with the transmitted half of wavefront 2, whose phase is φ2 +θ, and thus the interference intensity is (Born & Wolf, 1980, Chapter 7.2) either *I*_p_=*I*_1_/2 +*I*_2_/2 + (*I*_1_*I*_2_)^1/2^·cos(φ1 +π–φ2 –θ), denoted Case 1a, or *I*_p_=*I*_1_/2 +*I*_2_/2 + (*I*_1_*I*_2_)^1/2^·cos(φ1 +θ–φ2), denoted as Case 1b. Likewise, the reflected half of wavefront 2 either has phase φ2 +π or φ2 + 2θ, depending on where the reflection occurs, and when it interferes with the transmitted half of wavefront 1 at the secondary exit, whose phase is φ1 +θ, the interference intensity is either *I*_s_=*I*_1_/2 +*I*_2_/2 + (*I*_1_*I*_2_)^1/2^·cos(φ1 +θ–φ2 –π), denoted Case 2a, or *I*_s_=*I*_1_/2 +*I*_2_/2 + (*I*_1_*I*_2_)^1/2^·cos(φ1 –φ2 –θ), denoted Case 2b. Because of energy conservation, i.e. that *I*_1_+*I*_2_ must equal *I*_p_+*I*_s_, it is mandatory that Case 1a and 2b or Case 1b and 2a co-occur so that the cos(·) terms cancel out. In either case, it means the phases of the interference fringes at the primary and secondary exit port are different by π.

## References

[b1] Bertero M, Boccacci P (1998). *Introduction to Inverse Problems in Imaging*.

[b2] Born M, Wolf E (1980). *Principles of Optics: Electromagnetic Theory of Propagation, Interference and Diffraction of Light*.

[b3] Gustafsson MG (2000). Surpassing the lateral resolution limit by a factor of two using structured-illumination microscopy. J. Microsc.

[b4] Gustafsson MG, Agard DA, Sedat JW (1999). I^5^M: 3D widefield light microscopy with better than 100 nm axial resolution. J. Microsc.

[b5] Gustafsson MG, Shao L, Carlton PM (2008). Three-dimensional resolution doubling in widefield fluorescence microscopy by structured-illumination. Biophys. J.

[b6] Gustafsson MGL, Agard DA, Sedat JW (2000). Doubling the lateral resolution of wide-field fluorescence microscopy using structured-illumination. Proc. SPIE.

[b7] Hecht E (1998). *Optics*.

[b8] Heintzmann R, Cremer C (1999). Laterally modulated excitation microscopy: improvement of resolution by using a diffraction grating. Proc. SPIE.

[b9] Hell S, Stelzer EHK (1992). Properties of a 4pi confocal fluorescence microscope. J. Opt. Soc. Am. A.

[b10] Shao L, Isaac B, Uzawa S, Agard DA, Sedat JW, Gustafsson MG (2008). I^5^S: widefield light microscopy with 100-nm-scale resolution in three dimensions. Biophys. J.

